# Multiple arterial conduits for multi-vessel coronary artery bypass grafting in patients with mild to moderate left ventricular systolic dysfunction: a multicenter retrospective study

**DOI:** 10.1186/s13019-021-01463-5

**Published:** 2021-05-03

**Authors:** Hang Zhang, Wen Chen, Yang Zhao, Lichun Guan, Min Yu, Rui Wang, Xin Chen

**Affiliations:** 1 Department of Thoracic and Cardiovascular Surgery, Nanjing First Hospital, Nanjing Medical University, No. 68 Changle Road, Nanjing, 210006 China; 2 Department of Epidemiology and Biostatistics, School of Public Health, Nanjing Medical University, No. 101 Longmian avenue, Nanjing, 211166 China; 3 Department of Cardio-thoracic Surgery, Shanghai Eighth People’s Hospital, No. 8 Caobao Road, Shanghai, 200235 China; 4 Department of Cardiovascular Surgery, Shanghai General Hospital, Shanghai Jiao Tong University School of Medicine, No. 100 Haining Road, Shanghai, 200080 China

**Keywords:** Radial artery, Internal thoracic artery, Multiple arterial conduits, Coronary artery bypass grafting, Left ventricular systolic dysfunction

## Abstract

**Background:**

Advantages of multiple arterial conduits for coronary artery bypass grafting (CABG) have been reported previously. We aimed to evaluate the mid-term outcomes of multiple arterial CABG (MABG) among patients with mild to moderate left ventricular systolic dysfunction (LVSD).

**Methods:**

This multicenter study using propensity score matching took place from January 2013 to June 2019 in Jiangsu Province and Shanghai, China, with a mean and maximum follow-up of 3.3 and 6.8 years, respectively. We included patients with mild to moderate LVSD, undergoing primary, isolated multi-vessel CABG with left internal thoracic artery. The in-hospital and mid-term outcomes of MABG versus conventional left internal thoracic artery supplemented by saphenous vein grafts (single arterial CABG) were compared. The primary end points were death from all causes and death from cardiovascular causes. The secondary end points were stroke, myocardial infarction, repeat revascularization, and a composite of all mentioned outcomes, including death from all causes (major adverse events). Sternal wound infection was included with 6 months of follow-up after surgery.

**Results:**

243 and 676 patients were formed in MABG and single arterial CABG cohorts after matching in a 1:3 ratio. In-hospital death was not significantly different (MABG 1.6% versus single arterial CABG 2.2%, *p* = 0.78). After a mean (±SD) follow-up time of 3.3 ± 1.8 years, MABG was associated with lower rates of major adverse events (HR, 0.64; 95% CI, 0.44–0.94; *p* = 0.019), myocardial infarction (HR, 0.39; 95% CI, 0.16–0.99; *p* = 0.045) and repeat revascularization (HR, 0.42; 95% CI, 0.18–0.97; *p* = 0.034). There was no difference in the rates of death, stroke, and sternal wound infection.

**Conclusions:**

MABG was associated with reduced mid-term rates of major adverse events and cardiovascular events and may be the procedure of choice for patients with mild to moderate LVSD requiring CABG.

**Supplementary Information:**

The online version contains supplementary material available at 10.1186/s13019-021-01463-5.

## Introduction

Despite advances in multiple therapeutic strategies, left ventricular systolic dysfunction (LVSD) is still associated with poor clinical outcomes in patients suffering from coronary artery disease [1]. Surgical revascularization may provide survival benefit in selected patients. Several observational studies and the STICH (Surgical Treatment for Ischemic Heart Failure) trial have consistently reported the potential advantages of coronary artery bypass grafting (CABG) in prolonging survival and reducing cardiovascular events among patients with impaired left ventricular (LV) function [2–5]. Nevertheless, the choice of grafts and optimal grafting strategy remain uncertain, since patients with LVSD are in the status of long-lasting hypoperfusion and ventricular remodeling, thus requiring a long time for LV functional recovery after surgery [6].

Previous non-randomized studies have demonstrated the benefits to long-term survival of using 2 or more arterial conduits [7–9].. Theoretically, arterial conduits have superior patency rates and have been associated with a better prognosis in surgical revascularization. However, it would be argued whether impaired LV function could recover from this superiority, regarding patients with LVSD. A few investigations evaluating the long-term survival of multiple arterial CABG (MABG) versus single arterial CABG (SABG) in patients with LVSD came to different conclusions [10–12]. Besides, none of the studies did describe the specific cause of death which in many cases may be due to noncardiac factors and thus unrelated to graft choice. Herein, we conducted a multicenter, retrospective cohort study to evaluate the in-hospital and mid-term outcomes of MABG among patients with mild to moderate LVSD.

## Patients and methods

### Patients

Consecutive patients who received CABG from the 11 institutions in Jiangsu Province and Shanghai, China, from January 2013 to June 2019, were recruited from the Jiangsu Province CABG Registry (221.226.218.114,10,004/Multicenter) and Chinese Cardiac Surgery Registry-CABG section (ccsr.cvs-china.com). The two databases have similar parameter codes and are linked through the user’s identification. All data were de-identified before being delivered for analysis. No additional patient informed consent specific to this study was required given its retrospective nature. This study was approved by the ethics committee of Nanjing First Hospital (KY20170811–03).

### Study design

Patients were excluded if they met the following criteria: (1) concomitant procedures or prior cardiac surgery; (2) single vessel disease; (3) did not receive left internal thoracic artery (LITA); (4) hemodynamic instability that needed an intra-aortic balloon pump (IABP) or left ventricular assist device support before surgery; (5) urgent or emergency surgery, since these individuals were more likely to be performed with venous conduits. LV function was measured preoperatively with the two-dimensional echocardiography, assessed by 1–3 sonologists at each institute. Mild to moderate LVSD was defined as a left ventricular ejection fraction (LVEF) ≥ 30 / < 53% with reduction in global LV systolic function, in accordance with recently updated echocardiography guidelines [13]. Patients with LVEF ≥53% or < 30% were excluded from the analysis.

Eligible patients were with mild to moderate LVSD, undergoing primary, isolated, multi-vessel CABG and received at least LITA. Patients who received a LITA with either a radial artery (RA) or right internal thoracic artery (RITA), or both comprised the MABG group; patients who received a LITA supplemented by saphenous vein grafts (SVG) comprised the SABG group.

### Surgical procedure

The choice and combination of grafts were based on coronary artery anatomy, severity of coronary artery stenosis, and size of the grafted vessel. Internal thoracic artery grafts were predominately harvested with skeletonized technique. Various grafting strategies were used. These included conventional CABG with LITA anastomosed to left anterior descending artery (LAD) and SVG to other territories, use of RA grafts supplemented by SVG as clinically indicated, various LITA-right internal thoracic artery (RITA) configurations including free RITA, Y- or T-grafts, and single distal or sequential anastomosis techniques. All CABG procedures were performed by experienced surgeons at each institute. Standard methods for anesthesia, cardiopulmonary bypass, and myocardial protection were used according to local practice.

### Outcomes

The primary end points were death from all causes and death from cardiovascular causes. The secondary end points were: (1) major adverse events (MAEs), defined as a composite of death from all causes, stroke, myocardial infarction (MI) or repeat revascularization; (2) each individual MAEs event including stroke, MI, and repeat revascularization; and (3) sternal wound infection with 6 months of follow-up since surgical procedure. The tertiary outcomes included in-hospital events: postoperative mortality and morbidity. Patients were censored on January 31, 2020, and those who lost to follow-up were included using the last data recorded in the registries.

The standard intensive care unit (ICU) protocols were approximately similar in each institute, including ventilator support, sedation, and pain management. For postoperative variables, prolonged mechanical ventilation (PMV) was defined as a duration of postoperative mechanical ventilation or reintubation over 24 h [14]. Prolonged ICU stay was defined as a threshold of 48 h after transferring to ICU [15]. Death from cardiovascular causes included death caused by sudden cardiac death, MI, or heart failure. Stroke was defined as an incident ischemic or hemorrhagic cerebral event. MI included any subsequent visit for treatment of an incident acute MI. Repeat revascularization included any re-operative CABG or percutaneous coronary intervention (PCI) after surgery. Sternal wound infection was defined as an infected wound with coexisting osteomyelitis, dehiscence, or mediastinitis, or that requiring surgical debridement [16].

### Statistical analysis

Categorical variables are presented as numbers and corresponding percentages. Continuous variables are summarized with means ± standard deviations with normal distribution or medians (IQR) with skewed distribution. Differences in the characteristics between the treatment groups were assessed using chi-square test or Fisher’s exact probability method (categorical variables) and t-test or Mann-Whitney U-test (continuous variables) as appropriate. A 2-sided *P* value < 0.05 was considered statistically significant.

PSM was used to adjust for clinical baseline characteristics that were potentially confounding variables. Propensity scores were estimated using a multivariable logistic regression model, based on all baseline characteristics listed in Table 1. Patients treated with MABG were matched 1:3 to patients treated with SABG, using the propensity score with a nearest neighbor matching algorithm according to a caliper width of 0.02 without replacement. PSM was performed using the *MatchIt* package of R (version 3.5.3). Standardized mean differences (SMDs) were determined to compare baseline characteristics. A SMD ≤ 0.1 was deemed as an indicator of ideal balance between groups [17].

**Table 1:** Demographic characteristics of MABG vs SABG cohorts post-PSM

Characteristic	Overall (*n* = 919)	MABG (*n* = 243)	SABG (*n* = 676)	SMD
Age (years)	64.2 ± 9.0	63.2 ± 9.1	64.5 ± 8.9	0.09
BMI (kg/m^2^)	25.5 ± 3.1	25.7 ± 2.8	25.4 ± 3.2	0.1
eGFR (ml/min/1.73m^2^)	92.0 ± 26.3	93.3 ± 24.6	91.6 ± 26.9	0.06
LVEF (%)	46.0 (42.0, 49.0)	46.0 (42.0, 50.0)	47.0 (42.0, 49.0)	0.09
LVEF				0.05
30–40%	179 (19.5)	44 (18.1)	135 (20.0)	
41–52%	740 (80.5)	199 (81.9)	541 (80.0)	
LVEDV (mm)	53.5 ± 6.9	53.4 ± 6.7	53.5 ± 7.1	0.01
Male	750 (81.6)	198 (81.5)	552 (81.7)	0.01
Smoker	298 (32.4)	76 (31.3)	222 (32.8)	0.03
DM				0.02
No history	610 (66.4)	163 (67.1)	447 (66.1)	
NIDDM	226 (24.6)	58 (23.9)	168 (24.9)	
IDDM	83 (9.0)	22 (9.1)	61 (9.0)	
Hypertension	539 (58.7)	140 (57.6)	399 (59.0)	0.03
Carotid stenosis > 50%				0.05
None	763 (83.0)	200 (82.3)	563 (83.3)	
Unilateral	63 (6.9)	19 (7.8)	44 (6.5)	
Bilateral	93 (10.1)	24 (9.9)	69 (10.2)	
COPD	33 (3.6)	9 (3.7)	24 (3.6)	0.01
PVD	58 (6.3)	19 (7.8)	39 (5.8)	0.08
CVA	63 (6.9)	14 (5.8)	49 (7.2)	0.06
Dialysis	3 (0.3)	1 (0.4)	2 (0.3)	0.02
Prior MI	303 (33.0)	75 (30.9)	288 (33.7)	0.06
NYHA ≥3	531 (57.8)	134 (55.1)	397 (58.7)	0.07
Previous PCI	93 (10.1)	25 (10.3)	68 (10.1)	0.01
Mitral regurgitation				0.08
None	676 (73.6)	184 (75.7)	492 (72.8)	
Mild	200 (21.8)	50 (20.6)	150 (22.2)	
Moderate	39 (4.2)	8 (3.3)	31 (4.6)	
Severe	4 (0.4)	1 (0.4)	3 (0.4)	
No. vessel disease				0.04
2	103 (11.2)	25 (10.3)	78 (11.5)	
3	816 (88.8)	218 (89.7)	598 (88.5)	
Left main disease	269 (29.3)	61 (25.1)	196 (29.0)	0.08
Off-pump CABG	358 (39.0)	88 (36.2)	270 (39.9)	0.08

The variables are presented as mean ± standard deviation or median (IQR) or number (%). MABG multiple arterial bypass grafting, SABG single arterial bypass grafting, PSM propensity score matching, SMD standardized mean differences, BMI body mass index, eGFR estimated glomerular filtration (calculated by Modification of Diet in Renal Disease equation), LVEF left ventricular ejection fraction, LVEDV left ventricular end-diastolic volume, DM diabetes mellitus, NIDDM non-insulin-dependent diabetes mellitus, IDDM insulin-dependent diabetes mellitus, COPD chronic obstructive pulmonary disease, PVD peripheral vascular disease, CVA cerebrovascular accident, MI myocardial infarction, NYHA New York Heart Association, PCI percutaneous coronary intervention, CABG coronary artery bypass grafting

Time-to-event analyses were performed using Cox proportional hazards models to compare death from all causes and MAEs. Competing risk analyses (by treating death as a competing risk) were compared in the Fine and Gray models, regarding cumulative incidence of death from cardiovascular causes, stroke, MI, and repeat revascularization. The Fine and Gray models were constructed using the *cmprsk* package of R (version 3.5.3). Hazard ratios (HRs) with corresponding 2-sided 95% confidence intervals (CIs) were calculated and displayed. Data were analyzed using the R software (version 3.5.0, http://www.r-project.org/).

## Results

### Study population

**Fig. 1:**
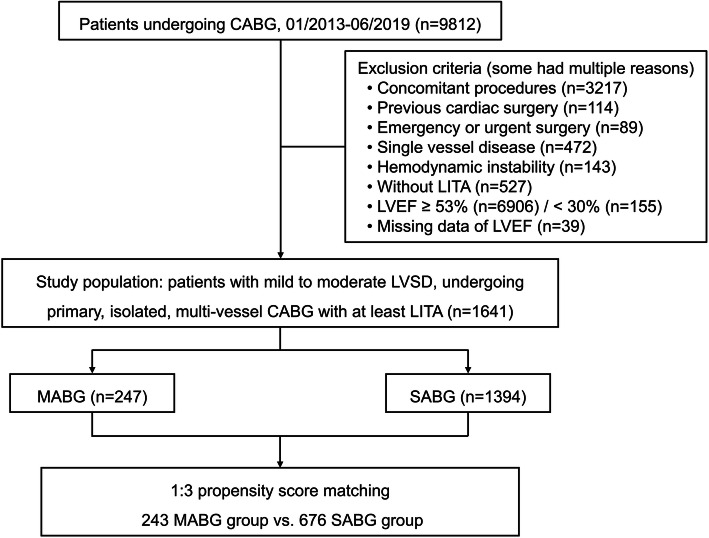
Flowchart showing patients included in the analysis. CABG, coronary artery bypass grafting; LITA, left internal thoracic artery; LVEF, Left ventricular ejection fraction; LVSD, left ventricular systolic dysfunction; MABG, multiple arterial bypass grafting; SABG, single arterial bypass grafting

From January 2013 to June 2019, a total of 1641 patients were eligible for investigation and treated with either MABG (*n* = 247) or SABG (*n* = 1394) (Fig. 1). Prior to matching, patients treated with MABG were younger, with a higher index of estimated glomerular filtration rate, and had lower proportions of hypertension, diabetes mellitus, and previous MI (all *p* < 0.05) (see Additional file 1). After PSM, patients were included in the MABG and SABG groups in a 1:3 ratio. All reported parameters were well balanced in 2 cohorts, with all baseline SMDs ≤0.1 (Table 1). Our data were highly complete with only smoker and cerebrovascular accident of covariates having less than 0.5% missing data. The 2 missing values were imputed to the most common category of binary variables. The median (IQR) follow-up time was 3.3 (1.8–4.8) years and 3.2 (1.8–4.6) years for MABG and SABG cohorts, respectively. Lost to follow-up occurred in 31 individuals (3.4%), and no significant difference was found between 2 cohorts (3.7% vs. 3.2%, *p* = 0.74).

### Arterial grafting strategies of MABG

All LITA grafts were in situ and used as single distal anastomosis directed to LAD in 94.3%, and to other arteries in 6.7%. For RA grafts, 71.5% were anastomosed to a single distal artery, 18.7% were used as sequential anastomoses, and 9.8% were performed as Y- or T-grafts. The RA grafts were used in most patients (67.2%) to bypass the left circumflex (LCX) region with target vessel stenosis > 70%, followed by right coronary artery (RCA) territory (8.5%). 71.2% of RITA grafts were in suit and used to bypass the RCA and LAD territories in approximately 34% of patients. In addition, 28.8% of RITA were available used as free grafts to mostly bypass the LCX and LAD territories. 33.6% of patients received total arterial grafts and 66.4% of patients received 2 or more arterial grafts with additional SVG.

### In-hospital outcomes

The operative and postoperative outcomes for the PSM cohorts are presented in Table 2. In the PSM cohorts, the total number of grafts were equal (3.5 ± 0.9 vs. 3.4 ± 0.8, *p* = 0.16). In-hospital death (1.6% vs. 2.2%, *p* = 0.78) and postoperative complication rates (PMV, prolonged ICU stay, IABP, stroke, acute MI, dialysis, red blood cell transfusion, sternal wound infection, reoperation for bleeding, LVEF at discharge, and length of stay) did not differ between 2 cohorts (all *p* > 0.05). Further, we compared unmatched in-hospital outcomes between the 2 groups, and found differences similar to the matched cohorts, with the exception of red blood cell transfusion (36.8% vs. 44.4%, *p* = 0.027) (see Additional file 1).

**Table 2:** Operative and postoperative in-hospital outcomes of the post-PSM MABG vs SABG cohorts

Outcome	Overall (n = 919)	MABG (*n* = 243)	SABG (*n* = 676)	*P*-value
No. of grafts	3.4 ± 0.8	3.5 ± 0.9	3.4 ± 0.8	0.16
RA	193 (21.0)	193 (79.4)	–	NA
RITA	114 (12.4)	114 (46.9)	–	NA
In-hospital death	19 (2.1)	4 (1.6)	15 (2.2)	0.78
PMV	188 (20.5)	46 (18.9)	142 (21.0)	0.49
PICUS	354 (38.5)	86 (35.4)	268 (39.6)	0.24
IABP	50 (5.4)	11 (4.5)	39 (5.8)	0.46
Stroke	6 (0.7)	2 (0.8)	4 (0.6)	0.70
Acute MI	8 (0.9)	2 (0.8)	6 (0.9)	0.93
Dialysis	12 (1.3)	3 (1.2)	9 (1.3)	0.91
RBC transfusion	385 (41.9)	91 (37.4)	294 (43.5)	0.10
Sternal wound infection	8 (0.9)	3 (1.2)	5 (0.7)	0.76
Reoperation for bleeding	12 (1.3)	2 (0.8)	10 (1.5)	0.66
LVEF (%)	47.0 (43.0, 50.0)	47.0 (44.0, 51.0)	47.0 (42.0, 50.0)	0.08
Length of stay (days)	20.0 (16.0, 24.0)	20.0 (16.0, 23.0)	19.0 (16.0, 24.0)	0.89

The variables are presented as mean ± standard deviation or median (IQR) or number (%). MABG multiple arterial bypass grafting, SABG single arterial bypass grafting, PSM propensity score matching, RA radial artery, RITA right internal thoracic artery, PMV prolonged mechanical ventilation, PICUS prolonged intensive care unit stay, IABP intra-aortic balloon pump, MI myocardial infarction, RBC red blood cell, LVEF left ventricular ejection fraction (estimated in patients who survival to discharge), NA not applicable

### Mid-term survival analysis

**Fig. 2:**
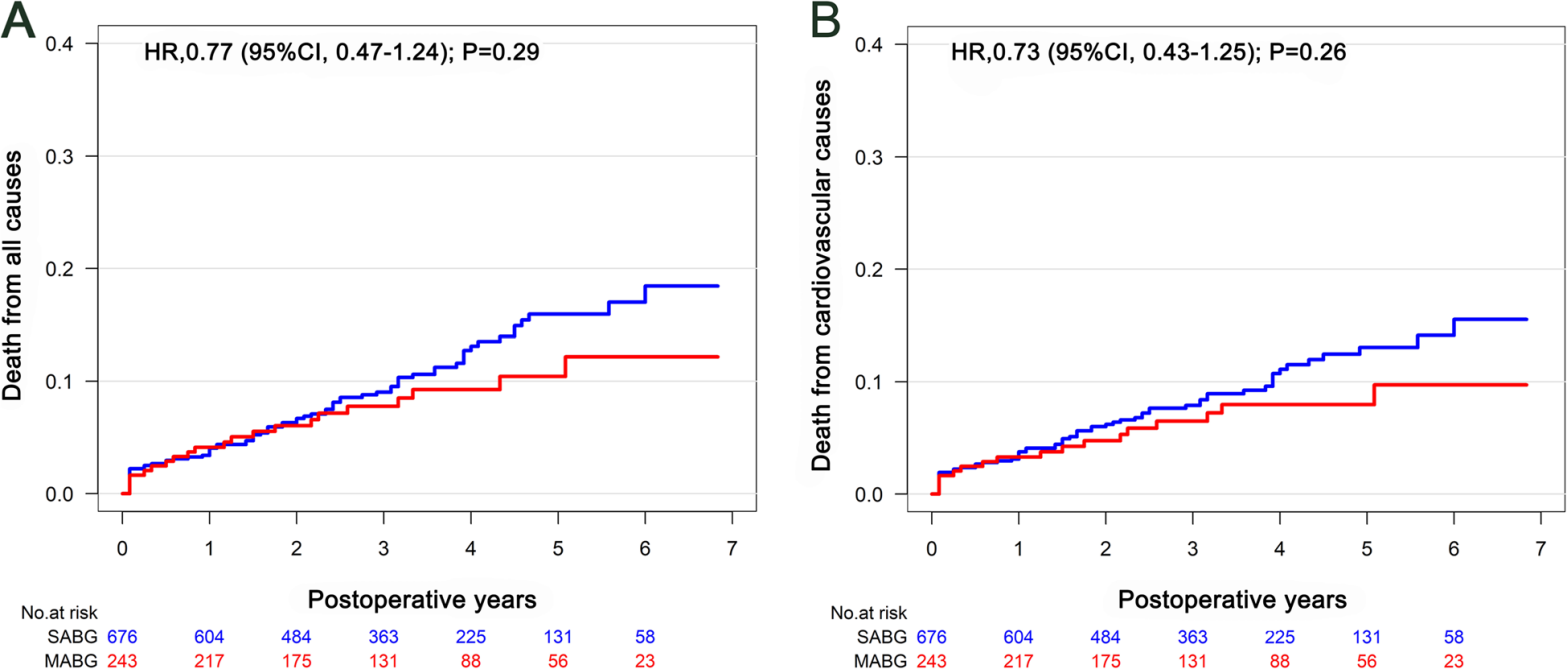
Cumulative incidence curves for the rates of death from all causes (**a**) and death from cardiovascular causes (**b**). HR, hazard ratio; CI, confidence interval; MABG, multiple arterial bypass grafting; SABG, single arterial bypass grafting

MABG was not associated with a significantly lower probability of death during follow-up period. Death from all causes for MABG was 12.2% (95% CI, 6.3–17.7%) versus 18.4% (95% CI, 13.3–23.3%) for SABG (HR, 0.77; 95% CI, 0.47–1.24; *p* = 0.29) (Table 3 and Fig. 2a). After adjusting for death from non-cardiovascular causes as a competing risk, death from cardiovascular causes for MABG was 9.7% (95% CI, 5.4–15.6%) versus 15.5% (95% CI, 11.1–20.6%) for SABG (HR, 0.73; 95% CI, 0.43–1.25; *p* = 0.26) (Table 3 and Fig. 2b).

**Table 3:** Follow-up outcomes of PSM MABG vs SABG cohorts

Outcome	MABG (n = 243)	SABG (n = 676)	HR (95% CI)	P-value
Death from all causes	12.2 (6.3–17.7)	18.4 (13.3–23.3)	0.77 (0.47–1.24)	0.29
Death from cardiovascular causes	9.7 (5.4–15.6)	15.5 (11.1–20.6)	0.73 (0.43–1.25)	0.26
MAEs	19.5 (12.2–26.1)	30.7 (24.8–36.1)	0.64 (0.44–0.94)	0.019
Stroke	4.0 (1.6–8.1)	4.7 (2.8–7.5)	0.91 (0.39–2.16)	0.84
MI	3.2 (1.1–6.9)	8.3 (5.4–11.8)	0.39 (0.16–0.99)	0.045
Repeat revascularization	4.1 (1.6–8.5)	10.2 (6.9–14.3)	0.42 (0.18–0.97)	0.034
Sternal wound infection^a^	2.1 (0.3–3.9)	1.0 (0.3–1.8)	0.50 (0.16–1.58)	0.24

^a^The event related to the period from surgical procedure to 6-month of follow-up; MABG multiple arterial bypass grafting, SABG single arterial bypass grafting, HR, hazard ratio, CI confidence interval, MAEs: major adverse events, MI, myocardial infarction

### Secondary outcomes

**Fig. 3:**
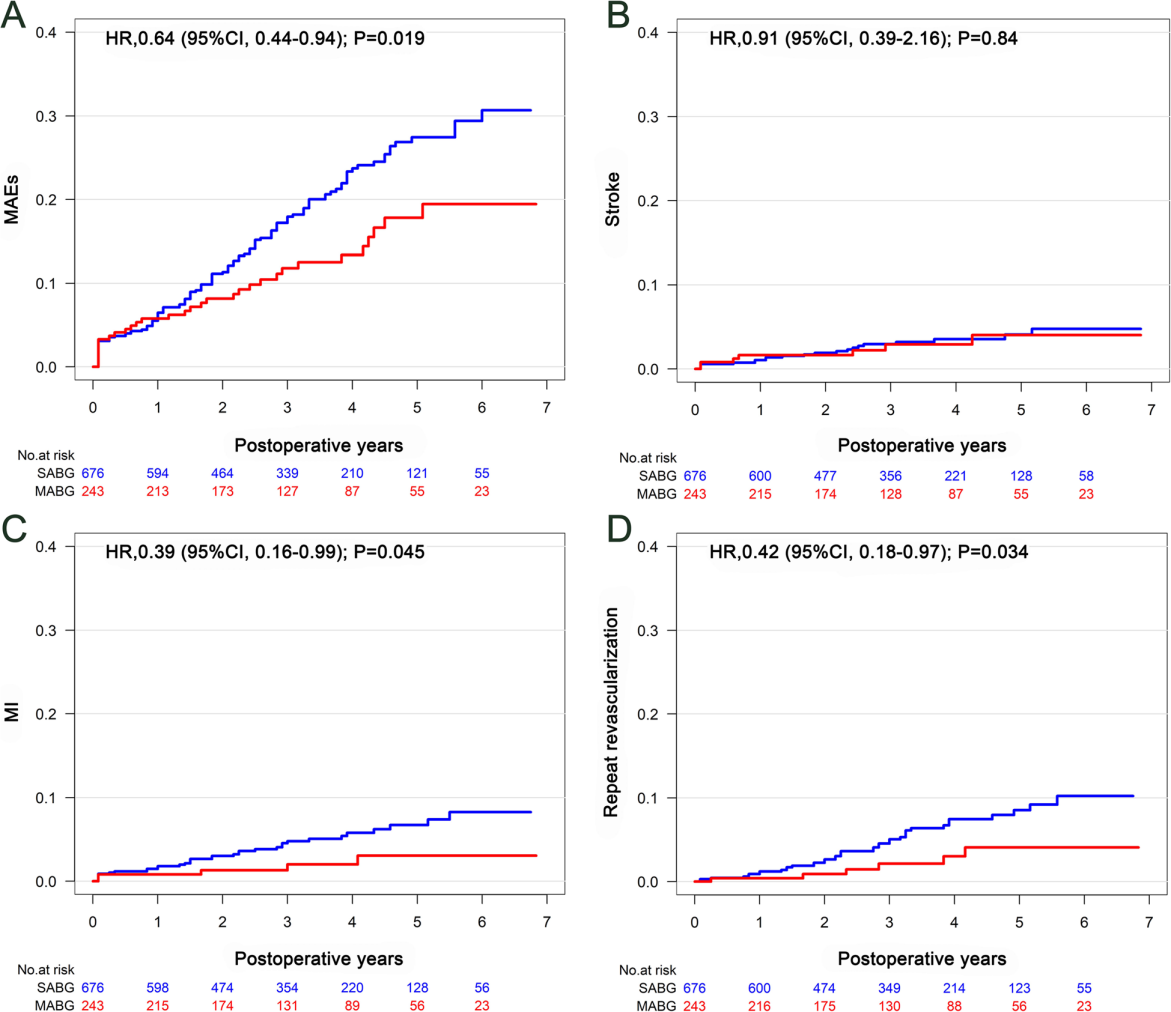
Cumulative incidence curves for the rates of MAEs (**a**), stroke (**b**), MI (**c**), and repeat revascularization (**d**). HR, hazard ratio; CI, confidence interval; MAEs, major adverse events; MI, myocardial infarction; MABG, multiple arterial bypass grafting; SABG, single arterial bypass grafting

The mid-term MAEs is presented in Table 3 and Fig. 3a. Fewer patients in MABG cohort experienced MAEs. The cumulative incidence of MAEs for MABG was 19.5% (95% CI, 12.2–26.1%) versus 30.7% (95% CI, 24.8–36.1%) for SABG (HR, 0.64; 95% CI, 0.44–0.94; *p* = 0.019). Stroke, MI, and repeat revascularization were compared in the Fine and Gray models. MABG was not associated with lower incidence of stroke compared with SABG (4.0% vs. 4.7%; HR, 0.91; 95% CI, 0.39–2.16; *p* = 0.84) (Table 3 and Fig. 3b). However, the cumulative incidence of MI was significantly lower in MABG cohort compared with SABG cohort (3.2% vs. 8.3%; HR, 0.39; 95% CI, 0.16–0.99; *p* = 0.045) (Table 3 and Fig. 3c). Likewise, the rate of repeat revascularization in MABG cohort was significantly lower than that in SABG cohort (4.1% vs. 10.2%; HR, 0.42; 95% CI, 0.18–0.97; *p* = 0.034) (Table 3 and Fig. 3d). The rate of sternal wound infection did not differ between 2 cohorts up to 6 months of follow-up after surgery (HR, 0.50; 95% CI, 0.16–1.58; *p* = 0.24) (Table 3).

### Sensitivity analysis

As a sensitivity analysis, we examined patients who received ≥3 grafts of MABG or SABG using similar PSM method. 203 and 566 patients were formed in MABG and SABG cohorts (data not shown). The rate of MAEs was statistically lower for MABG versus SABG (17.5% vs. 28.0%; HR, 0.64; 95% CI, 0.42–0.98; *p* = 0.036). Correspondingly, MABG was associated with a lower rate of repeat revascularization compared with SABG (3.5% vs. 9.6%; HR, 0.39; 95% CI, 0.15–0.99; *p* = 0.048). However, the incidence of MI showed no significant difference between MABG and SABG cohorts (2.9% vs. 8.0%; HR, 0.41; 95% CI, 0.14–1.16; *p* = 0.08). Death, stroke, and sternal wound infection did not differ between 2 cohorts (all *p* > 0.05) (see Additional file 1).

## Discussion

In this multicenter, population-based PSM study cohort evaluating the mid-term outcomes of MABG versus SABG in patients with LVSD, we found that (1) after PSM, in-hospital outcomes were similar between 2 cohorts; (2) MABG was associated with lower mid-term rates of MAEs and 2 individual components: MI and repeat revascularization; (3) MABG was not associated with reduced the mid-term rates of death and stroke; and (4) MABG did not increase the risk of sternal wound infection. These findings suggested that MABG should be importantly taken into consideration in patients with mild to moderate LVSD.

The threshold of LVSD defined in the previous guidelines was controversial [18, 19], mainly focusing on the argument of a borderline LVEF of 50–55%. Update European and American echocardiographic guidelines defined LVEF < 53% as abnormal, a threshold used in the present study [13]. Although multiple arterial grafting was much more complex and time-consuming than conventional SVG procedure, we did not observe worse in-hospital outcomes in MABG group. The excellent in-hospital outcomes of MABG may be probably related to the improvement in perfusion alteration, myocardial protection techniques, surgical skill, and perioperative care.

Patients with LVSD have different pathophysiological bases, most significantly in ventricular size, end-systolic pressure-volume, and chamber contractility [20]. Based on these changes, LVSD remained associated with diminished long-term survival despite much improvement was achieved in short-term outcomes after CABG [1]. The survival benefit derived from the LITA as a single arterial graft was reported in 1986 by Lytle et al. [21], and they also identified LITA as the conduit of choice in patients with low LVEF. The discovery promoted a transition from all SVG-CABG to the current standard of LITA/SVG-CABG in coronary surgery. Clinical practice guideline from Society of Thoracic Surgeons suggested that LITA should be used to bypass the LAD (class of recommendation [COR] I, level of evidence [LOE] B) and a second arterial graft (RITA or RA) should be considered in appropriate patients (COR II a, LOE B) [22].

Nevertheless, it remains unclear whether LVSD would negate any potential benefit of MABG, especially in severe cases. For the long-term outcomes, Galbut et al. [23] reported no survival benefit using bilateral internal thoracic artery (BITA) grafting in patients with LVEF < 30%. Similarly, Mohammadi et al. [11] did not find any BITA grafting benefit in late survival (mean, 8 years) in patients with LVEF < 40%. Schwann et al. [10] compared the long-term survival of RA-MABG versus SABG in the entire range of LVEF, and they found no survival difference in moderate to severe dysfunction (LVEF ≤35%) cohorts, but superior survival in mild dysfunction (LVEF = 36–50%) and normal cohorts (LVEF > 50%) of RA-MABG. Subgroup analysis from a recent population-based study showed that survival advantage of MABG was achieved in patients with moderate LVSD (LVEF = 35–50%), but that disappeared in patients with severe LVSD (LVEF < 35%) [7]. These researches potentially raised the topic of debate: should we perform MABG in the cases of LVEF < 35% or even < 30%? Given that approximately half of venous conduits occluded by 8–10 years post CABG [24], it appears to be more sufficient power to evaluate MABG benefit in patients who have a predicted survival of at least 10 years [25]. The vague recommendation that multiple arterial grafts be considered in “appropriate patients” or in patients “with reasonable life expectancy” offers little guidance to surgeons [22, 26]. We believe that patients with severe LVSD (LVEF < 30%) have severely impaired global systolic function and ventricular remodeling, acting as a powerful predictor of late mortality, and thus could not be considered as “with reasonable life expectancy”.

In our study, we excluded patients with severe LVSD, and a substantial proportion of patients were mild systolic dysfunction (median 46%, IQR 42–49%) with an average age of 64 years old. These patients may be clinically appropriate candidates for MABG and have a “reasonable life expectancy”. However, we failed to find a statistical difference in survival rate between MABG and SABG. The possible explanation was that we reported a small sample size study with inadequate follow-up time, making it underpowered to detect graft mediated difference in survival. Previous studies demonstrated that survival difference may start to appear 5 years after surgery in the general CABG population [9, 27], and patients with LVSD may take longer [28]. It is expected that disparities in graft patency should become manifest in terms of a difference in survival only in the large sample size and long-term following CABG.

Prior investigations suggested that the second arterial conduits affected survival similarly, regardless of RITA or RA was predominantly used [9, 29, 30]. In the present study, MABG was performed with several strategies including single distal or sequential anastomosis techniques, composite Y- or T-grafts, and total arterial revascularization. Our results concurred with findings from some other multicenter studies that MABG was less likely to undergo acute MI and repeat revascularization (PCI or surgical revascularization) [8, 31]. These recurrent ischemic events differences appeared earlier than death. There is clear evidence that failure of grafts to LAD adversely affects survival; however, failure of grafts to other target vessels is more likely to result in non-fatal cardiac events [32]. Despite BITA was identified as an independent risk factor for sternal wound infection [33], we did not observe a higher incidence of sternal wound infection in MABG. This discrepancy may be explained by the skeletonized technique used in our study, which harvests internal thoracic artery with preserving the lymphatic vessels and more of the blood supply to the sternum.

In our sensitivity analysis, there appeared to be more beneficial in SABG with increasing use of SVG, as reduction of disparities showed between 2 cohorts. Furthermore, there trended to decreased cardiovascular events in MABG, with the ratio of SVG increased. These findings were consistent with the study by Rocha et al. [34]. Whereas, further investigations are needed, as potential type II error may still come from a small sample size when creating subgroup analysis.

### Study limitations

Our study had several limitations. First, this was a retrospective study; therefore, potential biases could not be fully avoided, and a multicenter randomized controlled study with larger sample size and longer follow-up would add more weight to these results. Second, the selection bias of patients could not be fully avoided. Although we had excluded patients with severe LVSD (LVEF < 30%) and urgent states (emergency surgery or preoperative hemodynamic instability), arterial conduits used were at the discretion of surgeons. Surgeons would typically perform multiple arterial grafting if patients are expected to enjoy long survival after CABG. PSM cannot adjust for this. Moreover, surgeons’ expertise was also not taken into our consideration since that might affect the efficiency of treatment. Third, we were limited to obtain complete data of the computed tomography or coronary angiogram, and thus it was unclear that recurrent ischemic events occurred in grafts, treated vessels, or other coronary territories.

## Conclusion

The present study revealed that MABG was associated with lower mid-term rates of MAEs, MI, and repeat revascularization in comparison with SABG. These finding suggested MABG may be the procedure of choice for patients with mild to moderate LVSD requiring CABG. Additional adequately powered studies are required to further evaluate the long-term outcomes of MABG in patients with LVSD.

## Supplementary Information

**Additional file 1: Table S1** Demographic characteristics of MABG vs SABG groups before PSM. **Table S2** Operative and postoperative in-hospital outcomes of the before-PSM MABG vs SABG cohorts. **Table S3** Follow-up outcomes of PSM MABG vs SABG cohorts in patients received > 3 grafts. **Fig. S1** Cumulative incidence curves for the rates of death from all causes (A), death from cardiovascular causes (B), MAEs (C), stroke (D), MI (E), and repeat revascularization (F) in patients received > 3 grafts. HR hazard ratio, CI confidence interval, MAEs major adverse events, MI myocardial infarction, MABG multiple arterial bypass grafting, SABG single arterial bypass grafting.

## Data Availability

All data have been retrieved from the databases and are available from the corresponding author on reasonable request.

## Abbreviations

CABG: Coronary artery bypass grafting
LVSD: Left ventricular systolic dysfunction
LVEF: Left ventricular ejection fraction
MABG: Multiple arterial bypass grafting
SABG: Single arterial bypass grafting
LITA: Left internal thoracic artery
MAEs: Major adverse events
PSM: Propensity score matching
eGFR: Estimated glomerular filtration
DM: Diabetes mellitus
MI: Myocardial infarction
NYHA: New York Heart Association
PCI: Percutaneous coronary intervention
RA: Radial artery
RITA: Right internal thoracic artery
BITA: Bilateral internal thoracic artery
HR: Hazard ratio
CI: Confidence interval
