# Supplementary material for: Multiple arterial conduits for multi-vessel coronary artery bypass grafting in patients with mild to moderate left ventricular systolic dysfunction: a multicenter retrospective study
Source: J Cardiothorac Surg. 2021 May 3;16:123. doi: 10.1186/s13019-021-01463-5 (PMC8090915; doi:10.1186/s13019-021-01463-5)
Supplement: Supplementary file 1 — Additional file 1: Table S1 Demographic characteristics of MABG vs SABG groups before PSM. Table S2 Operative and postoperative in-hospital outcomes of the before-PSM MABG vs SABG cohorts. Table S3 Follow-up outcomes of PSM MABG vs SABG cohorts in patients received > 3 grafts. Fig. S1 Cumulative incidence curves for the rates of death from all causes (A), death from cardiovascular causes (B), MAEs (C), stroke (D), MI (E), and repeat revascularization (F) in patients received > 3 grafts. HR hazard ratio, CI confidence interval, MAEs major adverse events, MI myocardial infarction, MABG multiple arterial bypass grafting, SABG single arterial bypass grafting. [file 13019_2021_1463_MOESM1_ESM.docx]

**Supplementary material**

**Table S1** Demographic characteristics of MABG vs SABG groups before PSM

| Characteristic | Overall  (n = 1641) | MABG  (n = 247) | SABG  (n = 1394) | P-value | SMD |
| --- | --- | --- | --- | --- | --- |
| Age (years) | 64.6 ± 8.9 | 63.3 ± 9.5 | 64.8 ± 8.8 | 0.012 | 0.17 |
| BMI (kg/m^2^) | 25.6 ± 3.2 | 25.7 ± 2.8 | 25.6 ± 3.2 | 0.36 | 0.06 |
| eGFR (ml/min/1.73m^2^) | 90.3 ± 27.0 | 93.4 ± 24.5 | 89.7 ± 27.4 | 0.024 | 0.15 |
| LVEF (%) | 46.0 (41.0, 49.0) | 46.0 (42.0, 50.0) | 47.0 (41.0, 49.0) | 0.34 | 0.13 |
| LVEF |  |  |  | 0.14 | 0.11 |
| 30-40% | 351 (21.4) | 44 (17.8) | 307 (22.0) |  |  |
| 41-52% | 1290 (78.6) | 203 (82.2) | 1087 (78.0) |  |  |
| LVEDV (mm) | 53.5 ± 6.8 | 53.4 ± 6.6 | 53.5 ± 6.9 | 0.72 | 0.02 |
| Male | 1287 (78.4) | 202 (81.8) | 1085 (77.8) | 0.16 | 0.09 |
| Smoker | 492 (30.0) | 77 (31.2) | 415 (29.8) | 0.66 | 0.03 |
| DM |  |  |  | 0.015 | 0.21 |
| No history | 978 (59.6) | 167 (67.6) | 811 (58.2) |  |  |
| NIDDM | 452 (27.5) | 58 (23.5) | 394 (28.3) |  |  |
| IDDM | 211 (12.9) | 22 (8.9) | 189 (13.6) |  |  |
| Hypertension | 1035 (63.1) | 140 (56.7) | 895 (64.2) | 0.024 | 0.15 |
| Carotid stenosis > 50% |  |  |  | 0.59 | 0.07 |
| None | 1350 (82.3) | 203 (82.2) | 1147 (82.3) |  |  |
| Unilateral | 112 (6.8) | 20 (8.1) | 92 (6.6) |  |  |
| Bilateral | 179 (10.9) | 24 (9.7) | 155 (11.1) |  |  |
| COPD | 55 (3.4) | 10 (4.0) | 45 (3.2) | 0.51 | 0.04 |
| PVD | 115 (7.0) | 19 (7.7) | 96 (6.9) | 0.65 | 0.03 |
| CVA | 120 (7.3) | 14 (5.7) | 106 (7.6) | 0.28 | 0.08 |
| Dialysis | 9 (0.5) | 1 (0.4) | 8 (0.6) | 0.74 | 0.02 |
| Prior MI | 614 (37.4) | 76 (30.8) | 538 (38.6) | 0.019 | 0.17 |
| NYHA ≥ 3 | 1067 (65.0) | 153 (61.9) | 914 (65.6) | 0.27 | 0.08 |
| Previous PCI | 200 (12.2) | 26 (10.5) | 174 (12.5) | 0.39 | 0.06 |
| Mitral regurgitation |  |  |  | 0.82 | 0.07 |
| None | 1222 (74.5) | 186 (75.3) | 1036 (74.3) |  |  |
| Mild | 343 (20.9) | 52 (21.1) | 291 (20.9) |  |  |
| Moderate | 71 (4.3) | 8 (3.2) | 63 (4.5) |  |  |
| Severe | 5 (0.3) | 1 (0.4) | 4 (0.3) |  |  |
| No. vessel disease |  |  |  | 0.67 | 0.03 |
| 2 | 179 (10.9) | 25 (10.1) | 154 (11.0) |  |  |
| 3 | 1462 (89.1) | 222 (89.9) | 1240 (89.0) |  |  |
| Left main disease | 500 (30.5) | 62 (25.1) | 421 (30.2) | 0.11 | 0.11 |
| Off-pump CABG | 672 (41.0) | 91 (36.8) | 581 (41.7) | 0.15 | 0.09 |

The variables are presented as mean ± standard deviation or median (IQR) or number (%). MABG multiple arterial bypass grafting, SABG single arterial bypass grafting, PSM propensity score matching, SMD standardized mean differences, BMI body mass index, eGFR estimated glomerular filtration rate (calculated by Modification of Diet in Renal Disease equation), LVEF left ventricular ejection fraction, LVEDV left ventricular end-diastolic volume, DM diabetes mellitus, NIDDM non-insulin-dependent diabetes mellitus, IDDM insulin-dependent diabetes mellitus, COPD chronic obstructive pulmonary disease, PVD peripheral vascular disease, CVA cerebrovascular accident, MI myocardial infarction, NYHA New York Heart Association, PCI percutaneous coronary intervention, CABG coronary artery bypass grafting.

**Table S2** Operative and postoperative in-hospital outcomes of the before-PSM MABG vs SABG cohorts.

| Outcome | Overall  (n = 1641) | MABG  (n = 247) | SABG  (n = 1394) | P-value |
| --- | --- | --- | --- | --- |
| No. of grafts | 3.4 ± 0.8 | 3.5 ± 0.9 | 3.4 ± 0.8 | 0.11 |
| RA | 193 (11.8) | 193 (78.1) | - | NA |
| RITA | 118 (7.2) | 118 (47.8) | - | NA |
| In-hospital death | 31 (1.9) | 4 (1.6) | 27 (1.9) | 0.74 |
| PMV | 346 (21.1) | 47 (19.0) | 299 (21.4) | 0.39 |
| PICUS | 652 (39.7) | 87 (35.2) | 565 (40.5) | 0.12 |
| IABP | 90 (5.5) | 11 (4.5) | 79 (5.7) | 0.44 |
| Stroke | 8 (0.5) | 2 (0.8) | 6 (0.4) | 0.43 |
| Acute MI | 10 (0.6) | 2 (0.8) | 8 (0.6) | 0.66 |
| Dialysis | 23 (1.4) | 3 (1.2) | 20 (1.4) | 0.79 |
| RBC transfusion | 710 (43.3) | 91 (36.8) | 619 (44.4) | 0.027 |
| Sternal wound infection | 17 (1.0) | 4 (1.6) | 13 (0.9) | 0.33 |
| Reoperation for bleeding | 21 (1.3) | 2 (0.8) | 19 (1.4) | 0.48 |
| LVEF (%) | 47.5 (43.0, 50.0) | 47.0 (44.0, 51.0) | 48.0 (42.0, 50.0) | 0.58 |
| Length of stay (days) | 20.0 (16.0, 24.0) | 20.0 (16.0, 23.0) | 20.0 (16.0, 25.0) | 0.64 |

The variables are presented as mean ± standard deviation or median (IQR) or number (%). MABG multiple arterial bypass grafting, SABG single arterial bypass grafting, PSM propensity score matching, RA radial artery, RITA right internal thoracic artery, PMV prolonged mechanical ventilation, PICUS prolonged intensive care unit stay, IABP intra-aortic balloon pump, MI myocardial infarction, RBC red blood cell, NA not applicable.

**Table S3** Follow-up outcomes of PSM MABG vs SABG cohorts in patients received > 3 grafts.

| Outcome | MABG  (n = 203) | SABG  (n = 566) | HR  (95% CI) | P-value |
| --- | --- | --- | --- | --- |
| Death from all causes | 11.2 (5.0-17.0) | 15.7 (13.3-23.3) | 0.79 (0.46-1.36) | 0.39 |
| Death from cardiovascular causes | 9.3 (5.0-15.1) | 15.5 (11.1-20.6) | 0.69 (0.39-1.21) | 0.19 |
| MAEs | 17.5 (10.1-24.3) | 28.0 (22.5-33.1) | 0.64 (0.42-0.98) | 0.036 |
| Stroke | 4.9 (2.0-9.9) | 5.8 (3.5-9.1) | 0.89 (0.38-2.09) | 0.79 |
| MI | 2.9 (0.9-7.5) | 8.0 (5.1-11.8) | 0.41 (0.14-1.16) | 0.08 |
| Repeat revascularization | 3.5 (1.2-7.6) | 9.6 (6.6-13.4) | 0.39 (0.15-0.99) | 0.048 |
| Sternal wound infection^†^ | 2.5 (0.3-4.7) | 1.4 (0.4-2.4) | 0.50 (0.19-1.75) | 0.33 |

^†^The event related to the period from surgical procedure to 6-month of follow-up, MABG multiple arterial bypass grafting, SABG single arterial bypass grafting, HR hazard ratio, CI confidence interval, MAEs major adverse events, MI myocardial infarction.


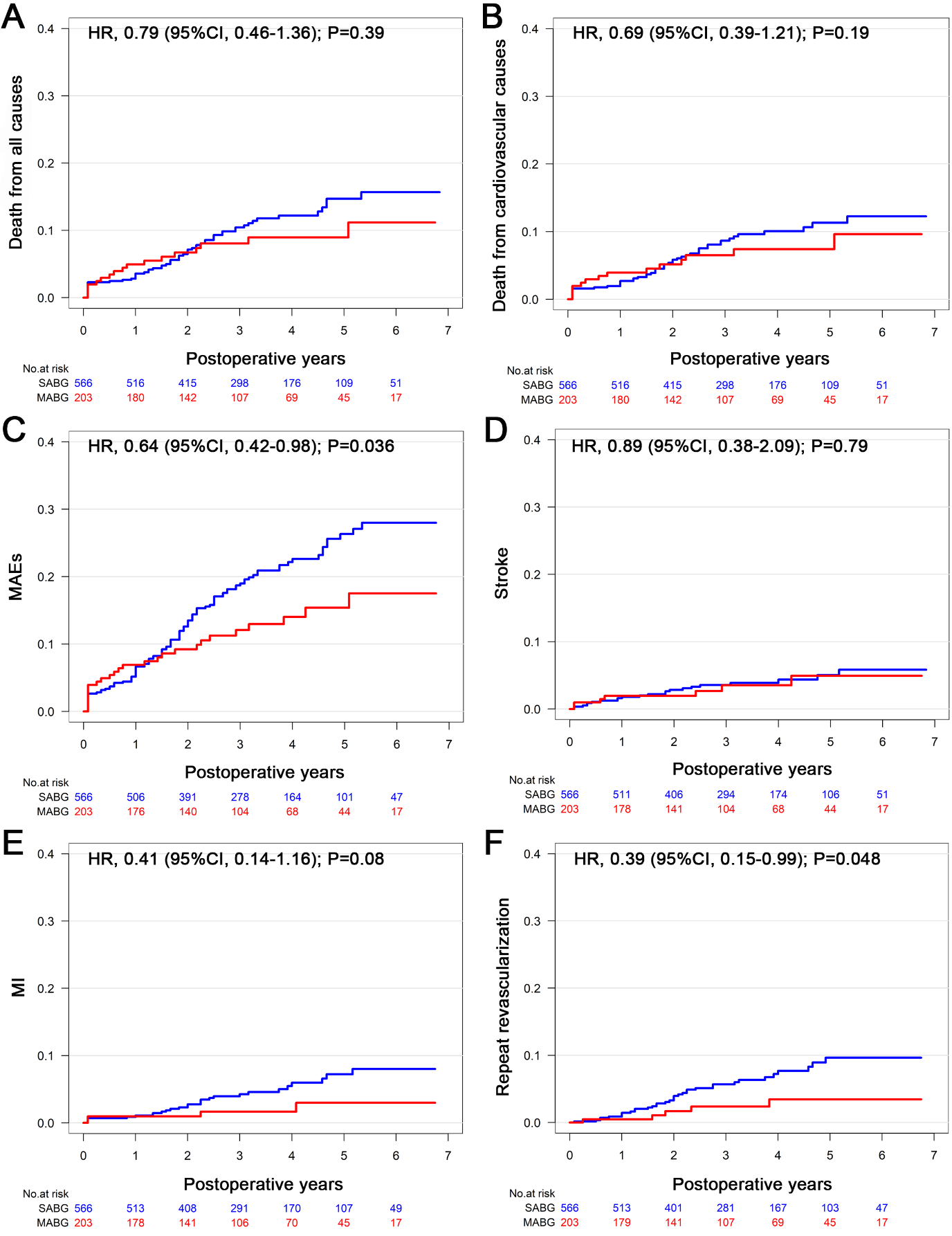


Fig. S1 Cumulative incidence curves for the rates of death from all causes (A), death from cardiovascular causes (B), MAEs (C), stroke (D), MI (E), and repeat revascularization (F) in patients received > 3 grafts. HR hazard ratio, CI confidence interval, MAEs major adverse events, MI myocardial infarction, MABG multiple arterial bypass grafting, SABG single arterial bypass grafting.
